# Clustered regulatory elements at nucleosome-depleted regions punctuate a constant nucleosomal landscape in *Schizosaccharomyces pombe*

**DOI:** 10.1186/1471-2164-14-813

**Published:** 2013-11-21

**Authors:** Ignacio Soriano, Luis Quintales, Francisco Antequera

**Affiliations:** Instituto de Biología Funcional y Genómica, Consejo Superior de Investigaciones Científicas (CSIC)/Universidad de Salamanca, Campus Miguel de Unamuno, 37007 Salamanca, Spain; Departamento de Informática y Automática, Universidad de Salamanca/Facultad de Ciencias, Plaza de los Caídos s/n, 37007 Salamanca, Spain

**Keywords:** Chromatin remodelling, Nucleosome dynamics, Transcriptional regulation, Genome organization

## Abstract

**Background:**

Nucleosomes facilitate the packaging of the eukaryotic genome and modulate the access of regulators to DNA. A detailed description of the nucleosomal organization under different transcriptional programmes is essential to understand their contribution to genomic regulation.

**Results:**

To visualize the dynamics of individual nucleosomes under different transcriptional programmes we have generated high-resolution nucleosomal maps in *Schizosaccharomyces pombe*. We show that 98.5% of the genome remains almost invariable during mitosis and meiosis while remodelling is limited to approximately 1100 nucleosomes in the promoters of a subset of meiotic genes. These inducible nucleosome-depleted regions (NDR) and also those constitutively present in the genome overlap precisely with clusters of binding sites for transcription factors (TF) specific for meiosis and for different functional classes of genes, respectively. Deletion of two TFs affects only a small fraction of all the NDRs to which they bind *in vivo*, indicating that TFs collectively contribute to NDR maintenance.

**Conclusions:**

Our results show that the nucleosomal profile in *S. pombe* is largely maintained under different physiological conditions and patterns of gene expression. This relatively constant landscape favours the concentration of regulators in constitutive and inducible NDRs. The combinatorial analysis of binding motifs in this discrete fraction of the genome will facilitate the definition of the transcriptional regulatory networks.

**Electronic supplementary material:**

The online version of this article (doi:10.1186/1471-2164-14-813) contains supplementary material, which is available to authorized users.

## Background

Nucleosomes play an essential role in the management of the eukaryotic genome by facilitating its packaging inside the nucleus. They also regulate basic genomic processes such as transcription, replication and recombination, either directly by controlling the physical access of regulators to DNA or indirectly by modulating their binding through a complex repertoire of histone modifications [1]. In recent years, DNA microarray and high-throughput sequencing technologies have enabled the mapping of nucleosomes at genome-wide scale in many organisms, including yeasts [2–6], *Drosophila*[7], *C. elegans*[8] and mammalian cells [9, 10]. In general, nucleosome profiles conform to a pattern where nucleosome-depleted regions (NDR) are present immediately upstream from the transcription start site (TSS) of most genes in the genome [11]. From these sites, regular nucleosome arrays are generated that extend into the transcribed regions. Since most of these studies have focused on the aggregated nucleosome profiles at the 5′ end of genes and transcribed units, the distribution of nucleosomes along intergenic regions has been comparatively less studied.

Recent analyses have revealed the extremely precise organization of nucleosomes at promoters to regulate the interaction between transcription factors and DNA. For example, periodic expression of the *CLN2* and *HO* genes during the *S. cerevisiae* cell cycle depends on the binding of their regulators to NDR in their promoters. Experimental manipulation of these promoters showed that when the binding sites were embedded in nucleosomes, transcription was still active but the cell cycle periodic expression was lost [12]. By contrast, other studies have shown that transcription factors such as Rap1 and Reb1 preferentially bind to their cognate sites on the surface of the nucleosome immediately upstream from the promoter NDR. The fact that the binding sites for Rap1 face outwards on the surface of the −1 nucleosome further highlights the relevance of their positioning and of their rotational symmetry in the regulation of gene expression [13].

How is this precise nucleosome organization generated and maintained? Poly (dA:dT) tracts can exclude nucleosomes [14, 15] and NDRs in *S. cerevisiae* are enriched in these elements [16]. Other sequences have also been selected to favour low nucleosome occupancy, such as those at the promoters of respiration genes, which are usually expressed in aerobic yeasts [17]. However, although some sequences favor nucleosome exclusion and others can promote nucleosome occupancy [15], the DNA sequence alone cannot specify the nucleosome profile observed *in vivo* at genome-wide scale [18, 19].

The statistical positioning model [20] proposed that nucleosome stacking against a physical barrier would passively generate regular arrays with an internucleosomal spacing inversely proportional to nucleosome density. Recent work has shown that nucleosome positioning in *S. cerevisiae* is an energy-dependent process, which in the presence of ATP and a cell extract can recapitulate the *in vivo* profile emanating from the 5′ NDRs, even at low nucleosome density and in the absence of transcription [16]. The model still requires the existence of physical barriers to act as organizing centers or focal points to set the beginning and confer directionality to the array [21, 22].

Nucleosome distribution in *S. pombe* has been comparatively much less studied than in *S. cerevisiae*. Several studies have generated genome-wide nucleosome maps using tiling microarrays or next generation sequencing. Some of these analyses have focused primarily on promoters and transcribed regions and have derived their conclusions from the aggregated nucleosome profiles of hundreds or thousands of genes [4, 5, 23, 24], while others have focused on the NDR profiles of replication origins and recombination hotspots [6, 25].

We have sequenced mononucleosomal DNA to a depth that allows a precise description of nucleosome dynamics at the level of individual nucleosomes across the *S. pombe* genome during mitosis and meiosis. We show that the great majority of the genome is organized in a very stable pattern of positioned nucleosomes and that NDRs overlap precisely with clusters of binding sites for transcription factors, which, in turn, could contribute to maintaining a regular nucleosome pattern across the genome.

## Results

### Widespread nucleosome positioning in the *S. pombe* genome

We have generated nucleosome maps in *S. pombe* by sequencing mononucleosomal DNA at a genome coverage ranging from 46- to 177-fold. This sequencing depth allows the high resolution mapping of individual nucleosomes by aligning sequence reads directly onto the reference genome followed by signal smoothing, with minimal mathematical modification of the raw data (see Methods and Additional file 1: Figure S1 and Additional file 2: Figure S2 for a comparison between raw and processed data and between the sequencing and hybridization analyses).

**Figure 1:**
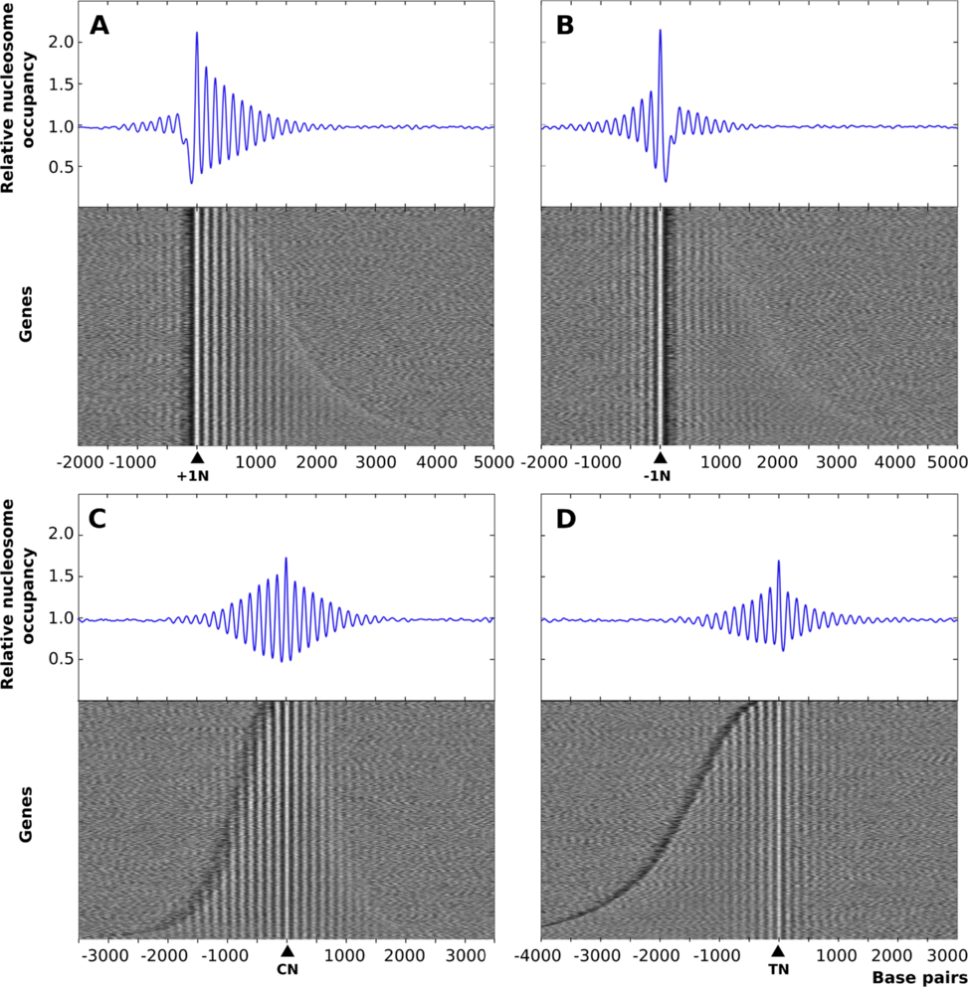
**Nucleosome profile of transcribed and intergenic regions in** ***S. pombe.*** The nucleosome profile of approximately 4000 *S. pombe* genes was aligned relative to the midpoint position of the +1 (+1 N) **(A)**, -1 (−1 N) **(B)**, central (CN) **(C)** and terminal (TN) **(D)** nucleosomes of each transcription unit. Genes are arranged by increasing size from top to bottom. Blue diagrams represent the relative nucleosome occupancy of the aggregated nucleosome profiles in the panels below. The data shown are from exponential mitotic diploid *pat1.114* cells.

To monitor the general distribution of nucleosomes along the genome, we selected the approximately 4000 genes where the transcription start site (TSS) has been annotated [4] and we aligned their nucleosomal profiles relative to the midpoint of the +1 nucleosome, which is the closest one downstream from the TSS (+1 N, Figure 1A, bottom). The resulting aggregated profile showed a maximum at +1 N that gradually declined towards the 3′ end of the transcribed regions (Figure 1A, top). This type of representation is the one most often used in the literature and generates comparable profiles in organisms as diverse as *S. cerevisiae*[2, 3, 21] and mammals [10].

Alignment to the midpoint position of +1 N generates sharper profiles than alignment to the TSS since +1 nucleosomes from different genes are perfectly aligned. Alignment to the TSS is expected to generate comparable nucleosomal profiles but of lower resolution due to variations in the distance between the TSS and the midpoint position of +1 N in different genes. This is reflected in the lower amplitude of the nucleosome peaks in the aggregated profiles. Alignment to TSS, however, is useful for establishing where transcription initiates relative to +1 N. Comparative alignments to +1 N and to TSS of *S. pombe* are shown in Additional file 3: Figure S3A.

**Figure 2:**
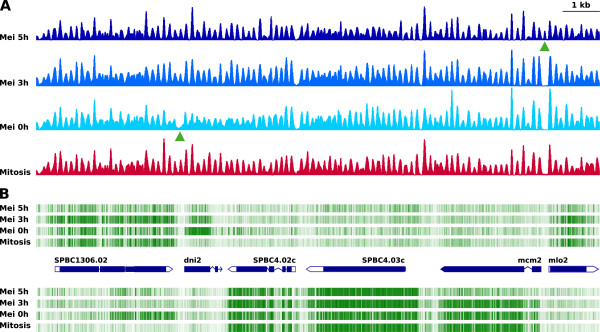
**Nucleosome dynamics and transcription during mitosis and meiosis. (A)**, Nucleosome patterns across 15 kilobases of the *S. pombe* genome from exponential diploid *pat1.114* cells (red) and from the same cells at the indicated times during synchronous meiosis (different shades of blue). Green arrowheads point to a nucleosome missing in meiosis at 0 h and to another present only in meiosis at 5 h. **(B)**, Strand-specific transcriptional profile generated by tiling microarrays. The intensity of the green lines correlates with the level of expression. Blue pointed rectangles represent genes where the coding (blue) and non-coding (white) regions are indicated. The absence or presence of the two nucleosomes in panel **(A)** (green arrowheads) correlates with the up- or down-regulation of the adjacent transcripts at 0 h and 5 h of meiosis, respectively.

The decline in the amplitude of the oscillations from the 5′ to the 3′ end of the genes in these profiles could result from the accumulative variation in the internucleosomal distances between genes and this would therefore not imply progressively lower nucleosome occupancy. This possibility was tested by using the nucleosome closest to the central position between the TSS and the transcription termination site (TTS) (central nucleosome, CN) as a reference for the alignment. The resulting symmetric profile (Figure 1C) indicated that nucleosomes at the 5′ and 3′ halves of transcription units were equally positioned, which is consistent with the relatively homogeneous pattern of nucleosome distribution along individual genes (Figure 2 and Additional file 4: Figure S4).

It has been reported that in *S. pombe*, unlike in *S. cerevisiae*, nucleosome positioning only occurs downstream from the 5′ NDRs [4, 24]. However, Figure 1A shows that alignment to nucleosome +1 generated a regular nucleosomal profile upstream from the NDR. The low amplitude of the peaks (indicative of higher variation in the internucleosomal distances) could be due to the different size of individual NDRs, which would cause the −1 nucleosome (−1 N, the closest upstream from the TSS) and those upstream from it to be out of phase between different genes. This possibility was confirmed by the finding that the alignment relative to -1 N generated a clear regular profile upstream from the NDR (Figure 1B). Alignment relative to -1 N was expected to blur the periodicity of nucleosomes downstream from the NDR generated when +1 N was used as a reference, as was indeed the case (compare Figure 1A and B). To test whether positioning was maintained downstream from transcribed regions, we used the terminal nucleosome (TN, the closest upstream from the TTS) as a reference for the alignment. Figure 1D shows that the resulting profile was virtually symmetric on both sides of TN indicating that nucleosome positioning extended beyond the TTS into the adjacent intergenic region. This symmetry, however, is not present in the nucleosomal profile of *S. cerevisiae*, due to the presence of a NDR at the 3′ end of many genes [26]. The comparative profiles generated by alignment to the TN or to the TTS in both yeasts is shown in Additional file 3: Figure S3C. Taken together, these results revealed a very high degree of genome-wide nucleosome positioning in *S. pombe* and indicated that aggregated nucleosome profiles vary depending on the reference used to align them and do not result from different properties of specific nucleosomes or gene regions.

### Nucleosome remodelling at meiosis-specific promoters

To address the point of how stable this pattern was under different transcriptional regimes, we searched for differences in the genomic distribution of nucleosomes in exponential vegetative cells and at different stages through meiosis. Specifically, we compared the nucleosome profiles of asynchronous haploid and diploid cells during mitosis and of diploid cells arrested in G0 (0 h) and at 3 and 5 hours after synchronous entry into meiosis, when chromosomes are recombining (3 h), and when the four haploid nuclei have separated and start maturing into spores (5 h) (see Methods). Previous studies have shown that several hundred genes are differentially transcribed during these early, medium and late stages [27–29] (see also Additional file 5: Table S1). Despite these differences, we found that the aggregated nucleosomal profile at these stages was indistinguishable from those seen during mitosis (See Additional file 6: Figure S5 for comparison of the profiles of mitosis and meiosis at 3 hours). In order to detect possible differences at the level of specific genes, we compared the nucleosomal profiles along the entire genome and found that the major differences between meiotic and vegetative cells were limited to 782 NDRs, which were present at 0, 3 or 5 h of meiosis but were absent in mitotic cells. We defined NDRs as regions spanning at least 150 nucleotides (corresponding to the eviction of at least one nucleosome. See Methods). As an example, Figure 2A shows a 15-kb region where a nucleosome at the 5′ end of the *dni2* gene in meiosis at 0 hours was absent while another occupied the NDR at the 5′ end of the divergently transcribed *mcm2* and *mlo2* genes in the 5-hour sample (green arrowheads). By contrast, all the other nucleosomes displayed a pattern of sharp, regularly spaced peaks that remained virtually invariable despite the different physiological stages and the chromosomal processes undergone by the cells. Since the average length of the 782 meiosis-specific NDRs was 214.68 +/− 71.06 nucleotides and the average distance between the mid-point of adjacent nucleosomes is 152 bp (Figure 1) [4, 25], we estimated that these NDRs were generated by the eviction of around 1100 nucleosomes. We have detected 78188 nucleosome peaks of nucleosome occupancy above the average genome-wide number of reads. Of these, we have considered 62824 nucleosomes (80.3%) as well positioned since their central coordinate was more than 100 nucleotides away from the nearest flanking peaks. These figures are consistent with the 77796 estimated number of nucleosomes obtained by dividing the 11825 kb of the *S. pombe* genome in http://www.pombase.org that remain after excluding rDNA, centromeric regions and constitutive NDRs by 152 bp. This means that nucleosome remodelling in the three meiotic samples that we analyzed relative to mitosis was limited to only 1.5% of the approximately 78000 nucleosomes in the *S. pombe* genome. The genomic position of the 2046 constitutive, 782 meiosis-specific and 26 mitosis-specific NDRs is indicated in Additional file 7: Table S2.

To monitor whether the meiosis-specific induced NDRs were associated with the differential transcription of the adjacent genes, we generated strand-specific transcription maps of exponential mitotic cells and of cells at 0, 3 and 5 hours during meiosis using tiling microarrays. The RNA level at these stages for all the annotated *S. pombe* genes (http://www.pombase.org) is indicated in Additional file 5: Table S1. Figure 2B illustrates that the eviction or repositioning of the two nucleosomes in the region shown coincided with the up- or down-regulation of the promoters of the *dni2* and *mcm2*/*mlo2* genes at 0 and 5 hours during meiosis, respectively. The nucleosomal and transcriptional profiles for the entire genome can be visualized in a searchable genome browser at http://genomics.usal.es/cgi-bin/gb2/gbrowse/Sp_nucdyn.

How widespread is the association between nucleosome remodelling and transcriptional regulation? Altogether, of the 782 meiosis-specific NDRs, 607 (77.6%) mapped to sites of transcription initiation. No transcripts were detected associated with the remaining 175 NDRs (32.4%) possibly due to a very low level of transcription, as has recently been shown for many genes in *S. pombe*[30]. Alternatively, these NDRs could be generated independently of transcription. They do not seem to be dependent on replication initiation either since only 20% of them colocalize with sites of binding for the Origin Recognition Complex (ORC) [6]. Out of the 607 NDRs that colocalized with TSS, 287 were adjacent to genes overexpressed at least 1.5-fold during meiosis relative to mitosis [27–29] (Additional file 5: Table S1).

To analyze the extent to which transcriptional activation was associated with promoter remodelling, we selected a total of 352 genes showing at least 4-fold differential expression during the following stages: 102 genes overexpressed in meiosis at 0 h relative to mitosis; 178 genes in meiosis at 3 h relative to 0 h; and 72 genes in meiosis at 5 h relative to 3 h [27–29] (Additional file 8: Table S3). Comparison of the nucleosomal profiles at these stages revealed that in 58/102 (0 h), 22/178 (3 h) and 23/72 (5 h) of the genes, differential expression was associated with the eviction of 1–2 nucleosomes from each promoter. This means that, altogether, a meiosis-specific NDR was generated in 103 out of the 352 promoters analyzed (30%) while the other 248 remained virtually identical despite the meiosis-specific overexpression. Of these 248, 118 did and 130 did not harbour constitutive NDRs. This indicates that the constant nucleosome profile present in the great majority of the genome is also maintained in approximately 70% of the promoters of meiosis-specific genes regardless of their differential activity.

### Nucleosome positioning along differentially expressed genes

**Figure 3:**
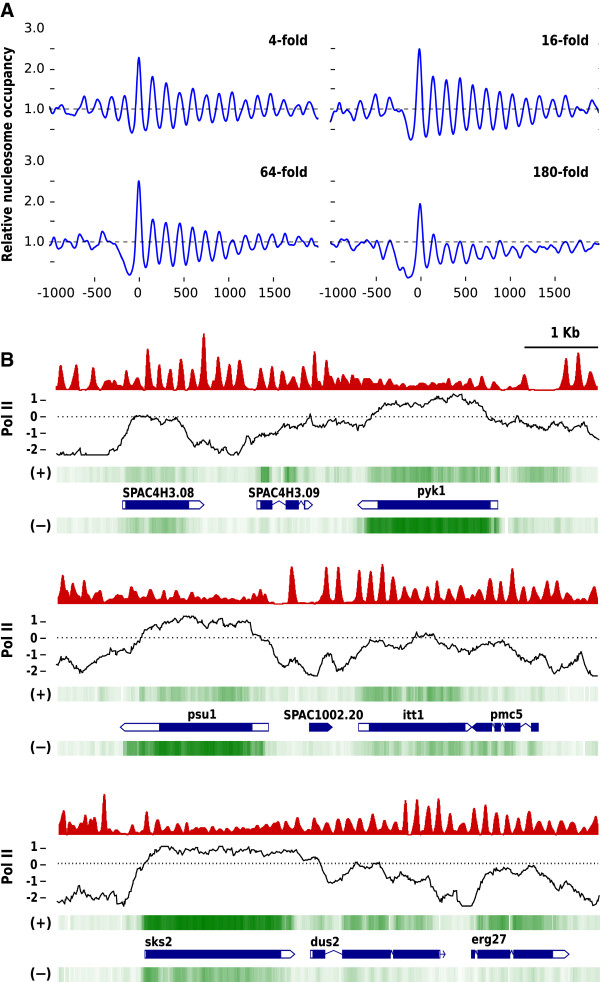
**Nucleosome positioning across differentially transcribed genes. (A)**, Relative nucleosome occupancy of four sets of 50 genes each with 4-, 16-, 64- and 180-fold expression levels relative to the background level during mitosis as detected by tiling microarray analysis. **(B)**, The positioned nucleosome profile of genes is lost in the *pyk1*, *psu1* and *sks2* genes, which are expressed over 180-fold relative to the background (see text for details). Transcription from both DNA strands (−) and (+) is shown. The profile of RNA polymerase II occupancy has been previously reported [32].

Our quantitative analysis of transcription in mitotic and meiotic cells revealed that the RNA levels between different genes ranged from undetectable to over 300-fold above the background (Additional file 5: Table S1). To address whether nucleosome positioning might be altered under conditions of high expression levels, we compared the nucleosomal profile of four different sets of 50 genes of similar length (approximately 1800 bp) whose RNA level during mitosis differed across a 50-fold range. Figure 3A shows that genes expressed 4- and 16-fold relative to the background maintained an identical nucleosomal pattern. Additional file 9: Figure S6 shows some examples of how the same nucleosomal profiles are maintained on the same genes expressed at different levels. In the case of genes with an RNA level 64-fold above the background, nucleosome positioning was slightly altered, as indicated by the small decrease in the height of the nucleosome peaks relative to genes with a lower level of expression. By contrast, nucleosome positioning was very much disturbed on genes expressed over 180-fold above the background. The RNA analysis indicated that only 59 (1.2%) of all the genes in the genome are expressed at this high level in mitosis (Additional file 5: Table S1) and hence their impact on the aggregated profiles of nucleosomes is negligible (Figure 1). NDRs, however, showed greater variability between the four different groups of genes since their average size increased with the level of expression (Figure 3A). Figure 3B illustrates the correlation between different RNA levels and the nucleosome organization of individual genes. For example, nucleosomes are well positioned across the *SPAC4H3.08* and *SPAC4H3.09* genes (top panel), which are expressed 3.4- and 32-fold above the background, but positioning is lost on the *pyk1* gene, which is expressed 238-fold. Positioning is also maintained along the *itt1* (10-fold) and *pmc5* (9.4-fold) genes (middle panel), but not on the *psu1* gene (180-fold). The same applies to the *dus2* (9.4-fold), *erg27* (21-fold) and *sks2* (254-fold) genes (bottom panel). Nucleosome delocalization on some genes showing high RNA levels is probably due to a high rate of nucleosome repositioning as a result of the high density of RNA polymerase II molecules crossing the gene [13, 31]. This possibility is supported by the strong correlation between genes expressed at a very high rate in exponential asynchronous cells, the loss of nucleosome positioning and a high level of RNA polymerase II occupancy in cells growing under the same conditions [32] (Figure 3B).

### Clusters of binding sites for transcription factors at nucleosome-depleted regions of meiosis-specific genes

**Figure 4:**
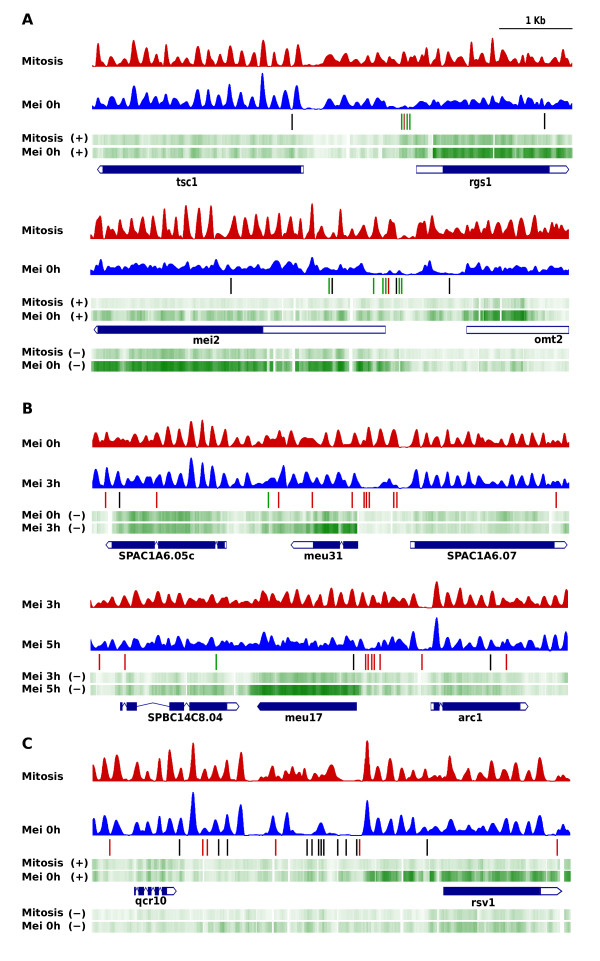
**Meiosis-specific NDRs overlap with clusters of binding sites for meiosis-specific transcription factors.** The mitosis and meiosis-specific expression pattern (green tracks) of the **(A)** *rgs1, mei2* and *omt2*, **(B)** *meu31* and *meu17 and* **(C)** *rsv1* genes is associated with meiosis-specific NDRs immediately upstream from their TSS. The nucleosomal profiles of the regions encompassing these genes during mitosis and at the indicated times of meiosis are shown. Sites of binding for the Ste11 (green), Mei4 (red) and Rsv1 (black) transcription factors are indicated by vertical lines.

Transcription factors have been implicated in the generation of NDRs through direct competition with nucleosomes for binding or, indirectly, through recruitment of chromatin remodellers [33, 34]. To explore the link between the binding of transcription factors and the specification of NDRs, we used the MEME algorithm (Multiple EM for Motif Elicitation) [35] to search for putative transcription factor binding sites (TFBS) in the NDRs of the 58, 22 and 23 meiosis-specific genes specifically transcribed at 0, 3 and 5 h mentioned above. By applying the same parameters and criteria as previously described [36] (see below and Methods) we identified three overrepresented motifs. The first of them was AACAAAG[AG]A, which is the binding site for the Ste11 transcription factor [37]. Ste11 activates the expression of cell type-specific genes (expressed in either M or P cells) as well as genes expressed in both M and P cells after nitrogen starvation [28]. Consistent with this, we identified 1 to 8 sites for Ste11 in 32 out of the 58 NDRs present in the sample of meiosis at 0 h, which was collected after 14 hours of culture in minimal medium without nitrogen (Figure 4A, green lines). By contrast, this motif was not enriched in the NDRs specific of 3 h and 5 h. Additional file 10: Figure S7A shows the overrepresentation of Ste11 binding sites in the NDRs of genes expressed at meiosis 0 h relative to the NDRs of genes expressed at 3 h and 5 h. The second motif identified was GTAAACAAA, which is the binding site for the Mei4 transcription factor. Mei4 regulates the induction of middle genes during meiosis [38–40], and it is also essential for the repression of early genes and for the activation of genes encoding transcription factors that, in turn, activate the expression of late genes [39]. This motif was present in 1 to 5 copies in 16 out of the 22 NDRs present at 3 h, and in 14 out of the 23 NDRs at 5 h (Figure 4B, red lines). Contrary to the TFBS of Ste11, the binding sites for Mei4 were not overrepresented in the NDRs of meiosis at 0 h (Additional file 10: Figure S7A). The third motif identified, CCCC[GTA]C, is the binding site for the transcription factor Rsv1 [41], and was present in 1 to 7 copies in 25 out of the 103 meiosis-specific NDRs, with no significant bias towards the 0 h, 3 h and 5 h samples (Figure 4C, black lines). Since the *ste11*, *mei4* and *rsv1* genes are expressed specifically in meiosis [28, 41, 42], these factors are likely candidates for being involved in the generation of NDRs harbouring clusters of binding sites for them.

As indicated above, 103 out of the 352 genes overexpressed over 4-fold during meiosis were associated with meiosis-specific NDRs while 118 genes harboured constitutive NDRs. Would binding sites for Ste11 and Mei4 also be overrepresented in such NDRs? MEME sequence analysis did not reveal enrichment in sites for Ste11 in any of them in the 0 h, 3 h and 5 h samples. The same analysis also showed that none of the genes overexpressed at 0 h relative to mitosis had any binding motifs for Mei4. However, 50% of the genes overexpressed at 3 h relative to 0 h had 1–2 motifs and 62.5% of the genes overexpressed at 5 h relative to 3 h had 1–3 motifs for Mei4. These results are consistent with those presented in Additional file 10: Figure S7 and imply that this factor is likely to be also involved in the specific expression of the middle and late meiotic genes through the binding to those NDRs that are constitutively present at the 5′ end of some genes specifically expressed during meiosis.

### Clusters of binding sites for transcription factors at nucleosome-depleted regions of cell cycle and stress response genes

**Figure 5:**
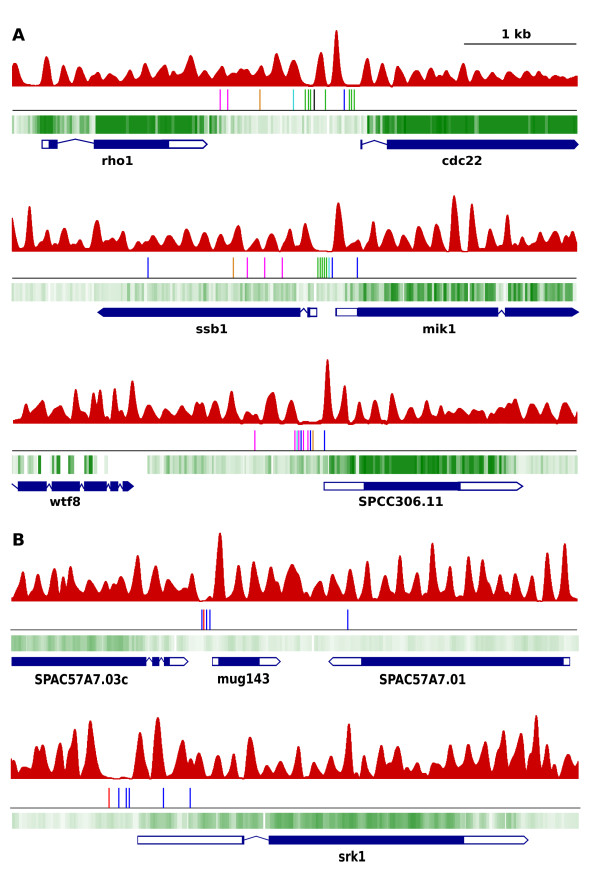
**NDRs of cell cycle-regulated and stress response genes overlap with different clusters of binding sites for transcription factors. (A)** The *cdc22*, *mik1* and *SPCC306.11* genes are periodically expressed during the mitotic cell cycle. Clusters of binding sites for six different transcription factors [36] are indicated by vertical lines: (purple, Ace2; dark blue, FKH; green, MBF; black, Dbl10; orange and light blue, overrepresented motifs not associated to previously identified factors. **(B)** The *mug143* and *srk1* genes are overexpressed under oxidative stress. Binding sites for two transcription factors are indicated (see text for details). The nucleosome profile across the five regions in mitotic cells is shown on top of each diagram (red). The transcribed DNA strand during mitosis in the absence of stress corresponding to the five genes described above is shown (green).

To address how general the colocalization of NDRs with clusters of TFBS for genes not specific for meiosis was, we took the reverse approach of testing whether previously identified clusters colocalized with NDRs. Oliva et al. [36] described 34 clusters of putative binding sites for 6 transcription factors including Ace2, FKH, MBF and DBL10, upstream from *S. pombe* cell cycle-regulated genes. We overlapped the position of the TFBS with the nucleosome profile of mitotic cells and found that in 31 out of the 34 cases (91.2%) the clusters showed a striking overlap with NDRs (Figure 5A). The TFBS present in them were different from those identified in the meiosis-specific NDRs (Figure 4), indicating that NDRs of different functional classes of genes encompassed clusters of different TFBS. To further test this association, we selected the promoters of the 36 genes showing the highest overexpression under conditions of oxidative stress in *S. pombe* that contained an NDR in their promoters [43, 44]. MEME analysis revealed that 19 NDRs included 1 to 3 copies of the consensus CRE element (TGACGT) (Figure 5B, blue lines) and 14 NDRs had 1 to 2 copies of the CRE variant element TGACATCAT [45] (Figure 5B, red lines). These sequences are bound by proteins of the ATF CREB that play an important role in stress response and that in *S. pombe* are encoded by the *atf1*, *pcr1*, *atf21* and *atf31* genes [46–48]. Additional file 10: Figure S7B shows the overrepresentation of these two sequence elements in the NDRs of the 36 stress response genes.

### Transcription factors contribute redundantly to maintaining NDRs

**Figure 6:**
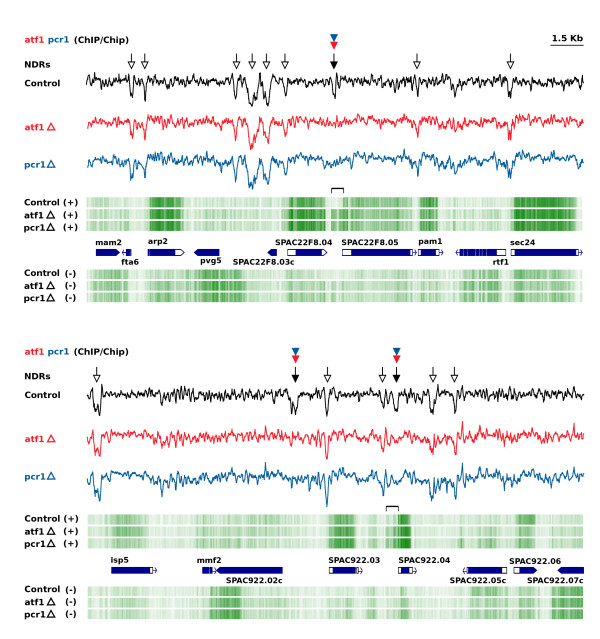
**Transcription factors are required for the maintenance of NDRs.** Nucleosomal and NDR patterns generated by tiling microarrays of control cells (972 h-) and of *atf1*Δ and *pcr1*Δ mutants are represented by black, red and blue lines, respectively. Sites of Atf1 and Pcr1 binding mapped by ChIP/Chip [49] are indicated by red and blue arrowheads. Black arrows indicate NDRs that disappear in the absence of Atf1 or Pcr1 and white arrows point to NDRs not bound by Atf1 and Pcr1 that remain invariable in the three strains. Strand-specific transcription profiles for the three strains are shown in green. Alternative transcription start sites of the *SPAC22F8.05* (top diagram) and *SPAC922.04* (bottom diagram) genes associated with the closing of NDRs dependent on Atf1 and Pcr1, are indicated by brackets.

The precise colocalization of NDRs and clusters of TFBS suggested the possibility that combinations of different transcription factors could contribute to the generation or maintenance of NDRs. To address whether actual binding was required, we independently deleted the genes encoding the Atf1 and Pcr1 transcription factors and monitored the impact on the genome-wide pattern of NDRs by hybridizing mononucleosomal DNA from the two mutant strains to genomic tiling microarrays. These two factors form a heterodimer that regulates the expression of the core environmental stress response genes and also of other genes, and we chose them for this analysis because their binding sites in the genome have been determined [49]. We overlapped their genomic distribution with the nucleosome profile of mitotic wild-type cells and found that of the 148 major sites of Atf1 and Pcr1 binding in the genome in the absence of stress, 116 (78.4%) were located in NDRs. However, we also found that only 22 out of the 116 (19%) NDRs disappeared in both mutant strains (Figure 6). In these 22 cases, 6 and 3 genes were over- and underexpressed, respectively (Additional file 11: Table S4). In some of the cases where the level of expression remained invariable, such as upstream from the *SPAC22F8.05* (Figure 6, top) and *SPAC922.04* genes (Figure 6, bottom), the transcription start site was shifted approximately 500 bp upstream relative to wild-type cells. In the *SPAC922.04* gene, transcription initiated in a NDR that remained open in the mutant cells but that was not used for transcription initiation in wild-type cells.

The other 94 (81%) NDRs that remained unaltered in the *atf1*Δ and *pcr1*Δ mutants were probably maintained by other transcription factors that bind to the same NDRs, consistent with the presence of multiple binding sites for TFs in NDRs of meiosis-specific and cell cycle-regulated genes (Figures 4 and 5). A possible candidate to contribute to the maintenance of these NDRs could be the Php4 CCAAT-binding factor [50] since analysis of the 116 NDRs bound by Atf1/Pcr1 showed that the CCAATCA sequence was present in half of the 94 NDRs that remained unaffected but was absent in the 22 that dissappeared in *atf1*Δ and *pcr1*Δ cells. It is also possible that Atf21 and Atf31, another two members of the ATF CREB family of transcription factors, could functionally replace Atf1 and Pcr1 in some promoters [48]. This is supported by the fact that of the 148 genes immediately downstream from the NDRs bound by Atf1 and Pcr1 in the genome, only 18 and 14 genes were overexpressed and 11 and 16 genes were underexpressed more than 2-fold in the *atf1*Δ and *pcr1*Δ mutants, respectively (Additional file 12: Table S5). The transcriptional profile for the entire *S. pombe* genome in wild-type, *atf1*Δ and *pcr1*Δ mutant cells is shown in the genome browser linked to this article.

## Discussion

### Nucleosome dynamics of the *S. pombe* genome

As a consequence of the high resolution of the maps generated by us here, our results regarding the nucleosomal organization in *S. pombe* differ in several aspects from previous studies. For example, it has been proposed that nucleosomal positioning would coincide with the length of the transcribed units and that it is absent in inactive genes, suggesting an active role for transcription in positioning [4]. However, we observed that nucleosomal positioning extended beyond the transcription termination site (Figure 1D) and that it was also present at genes showing low or undetectable transcription levels (Figure 3A, B and Additional file 9: Figure S6). Also, although previous analyses have failed to detect positioning upstream from the NDR at the 5′ position of the genes [4, 24, 25], we found that nucleosomal arrays emanate bidirectionally from this NDR (Figure 1B) in a fashion comparable to that in *S. cerevisiae*[2, 3, 26]. A comparison between the nucleosomal profiles of *S. pombe* and *S. cerevisiae* using the same criteria shows that profiles downstream from +1 N were similar (Additional file 3: Figure S3A). However, nucleosome positioning upstream from the NDR was slightly higher in *S. cerevisiae* (as indicated by the height of the peaks). To test whether this effect might be partially due to the fact that the size of NDRs is more homogeneous in *S. cerevisiae*[16, 26] than in *S. pombe*[4], we also aligned the nucleosomal profiles to -1 N and obtained comparable results in both yeasts (Additional file 3: Figure S3B). In the two cases, positioning was stricter downstream (+1 N alignment) than upstream (−1 N alignment) from the NDR, probably due to the variable distance of other upstream NDRs and genes that set the nucleosomal arrays out of phase in the aggregated profiles.

It is possible that the regular nucleosome pattern upstream from the NDR in *S. pombe* was not detected earlier owing to the limited resolution of the microarray analyses and the lower sequencing depth of previous studies, which made it difficult to define the position of the −1 nucleosome [4, 5, 24, 25].

Another contribution of our work is the possibility of a genome-wide quantitative assessment of nucleosome dynamics. We estimate that only 1.5% of the approximately 78000 nucleosomes in the genome are remodelled during the expression of the meiotic transcriptional programme. Remodelling, however, is not always associated with transcriptional activation, as illustrated by the invariable nucleosome profile of 70% of the promoters of the 352 genes overexpressed more than 4-fold at specific stages of meiosis relative to mitosis (Additional file 8: Table S3). A lack of correlation between transcriptional activity and the presence of NDRs at promoters has also been observed in *S. cerevisiae*[26].

### Specification of genomic nucleosomal patterns

In *S. cerevisiae*, poly (dA:dT) elements are overrepresented in the NDRs associated with promoters [14, 15]. By contrast, these elements are not particularly enriched in *S. pombe* and, in agreement with previous data [4], we found that only 8.3% and 3.4% of the poly (dA:dT) elements 15 and 7 nucleotides long, respectively, colocalized with constitutive NDRs. Conversely, only 13% of these included poly (dA:dT) tracts 7 or 15 nucleotides long (data not shown). These results suggest that the contribution of these elements to the generation of NDRs is much smaller than in *S. cerevisiae*[14–16].

The statistical positioning model proposes the existence of physical barriers from which regular nucleosomal arrays are generated [20, 21] and transcriptional regulatory complexes are obvious candidates for this role. These large complexes include transcription factors, coactivators, histone modifiers and chromatin remodellers [51] and are targeted to promoters by transcription factors, which are their only components capable of recognizing specific sequences on DNA. Binding motifs for transcription factors are made up of degenerated sequences 6–10 bp long scattered across the genome, although only a small fraction of them are actually bound *in vivo*. For example, in *S. cerevisiae* the Leu3 transcription factor binds only a subset of all potential binding sites in the chromosomes even though the protein binds to all of them with comparable affinity on naked DNA. Sites bound *in vivo* were strongly correlated with a low nucleosome occupancy, which was not dependent on the presence of the Leu3 protein, suggesting an opportunistic use of accessible chromatin sites already available in the genome [52]. In *S. pombe*, 148 (6.9%) out of the 2141 TGACGT hexamers representing potential binding sites for Atf1/Pcr1 are detectably bound in the genome [49]. Of these 148 sites, 116 (78.4%) colocalized with NDRs, of which only 22 disappeared in the absence of the factors (Figure 6), pointing to the presence of redundant elements in their maintenance, as in the case of Leu3. This possibility is strongly supported by the recent finding that up to 8 proteins are involved in the maintenance of the NDR at the *CLN2* promoter in *S. cerevisiae*[53]. Along the same lines, NDRs at heterochromatic regions in *S. pombe* depend on the combinatorial contribution of at least 5 different proteins [54]. However, NDRs can also be generated as a consequence of the binding of a single transcription factor, as in the case of the *M26* mutation in *S. pombe*, which generates a binding site for Atf1 [55]. Also, DNA sequences refractory to bending such as poly (dA:dT) [14, 15], poly G tracts [5] and sequences at some promoters [17] favour the exclusion of nucleosomes.

Previous studies have found that the binding sites for transcription factors tend to cluster at promoters [3, 36, 56, 57] and our results show that these clusters overlap precisely with NDRs at the promoters of meiosis-specific (Figure 4), cell cycle-regulated genes, and stress-response genes (Figure 5). The complexity of the clusters of binding sites for TFs suggests that each NDR and each promoter is probably unique, making it very difficult to predict the effect that the removal of specific elements will have in its maintenance or on the expression of the adjacent genes. For example, while the lack of Leu3 in *S. cerevisiae*[52] and of Atf1/Pcr1 in *S. pombe* (Figure 6) has a limited impact on NDRs genome-wide, deletion of the Abf1 and Reb1 transcription factors in *S. cerevisiae* negatively affects a larger number of NDRs [33, 57]. Similarly, the Sap1 protein, which is involved in DNA replication and mating-type switching and binds preferentially to NDRs, is required for the maintenance of a large number of them in *S. pombe*[5].

This collaborative strategy of TFs to assemble transcription complexes at promoters has several immediate advantages for genome regulation. The first is that it favours transcription initiation from the 5′ end of genes and reduces spurious initiation at single binding sites dispersed along the genome that are usually occluded by nucleosomes. Second, the redundant contribution of several factors guarantees the maintenance of NDRs, regardless of fluctuations in their concentrations. Altogether, we have identified 2046 constitutive NDRs in mitotic and meiotic cells and 782 meiosis-specific NDRs. The constitutive presence of a large fraction of NDRs could facilitate the rapid transcriptional induction of, for example, stress response genes [44], and could also contribute to other processes such as the specification of meiotic recombination hotspots [6, 58]. A third function of NDRs and the complexes bound to them would be to act as barriers [20] or organizing centers from where nucleosome arrays are generated [16, 21, 22]. The targeting of these complexes by transcription factors to the same specific sites in every genome and the relatively close proximity between promoters could explain why a virtually identical nucleosomal arrangement is maintained in all the cells in the population under very different physiological conditions. A stable nucleosomal pattern during mitosis and meiosis has also been observed in *S. cerevisiae*[59]. This is compatible with an active turnover of nucleosomes as has been described in *S. cerevisiae*[60] and with variations in the epigenetic modifications of histones [61] that could provide flexible regulatory signalling while maintaining a constant nucleosomal framework.

## Conclusions

Nucleosome dynamics in *S. pombe* genome is limited to a very small fraction of the genome that overlaps with regulatory regions. We have shown that NDRs encompass clusters of TFBS specific for different gene functions such as meiosis, cell-cycle regulation and stress response. Sequence analysis of these narrow and well-defined regions should help to define genomic regulatory networks based on the combinatorial and collective contribution of regulatory elements shared by different promoters. On the other hand, the high degree of order at nucleosome level probably underlies the ordered structure of chromatin organization at higher levels, evidenced by the specific pattern of interactions between different chromosomes [62] and by the global three-dimensional architecture of the nucleus, where each chromosome occupies a specific territory [63, 64]. Despite the many instances in which nucleosome remodelling at promoters is not associated with changes in transcriptional activity [26], the maintenance of a highly organized nucleosomal pattern is likely to be important for genomic stability, as illustrated by the gross alterations in recombination [65], and for preventing cryptic and unscheduled antisense transcription that results when the level of histones or the positioning of nucleosomes is altered [66–68].

## Methods

### *S. pombe* strains, growth conditions and meiosis synchronization

Wild-type (972 h-), *atf1Δ* (h + ura4.d18 leu1.32 ade6M210 atf1Δ::kanMX4) and *pcr1Δ* (h + ura4.d18 leu1.32 ade6M210 pcr1Δ::kanMX4) cells were grown in rich medium (YES) at 32°C up to a A595 = 0.8. Diploid pat1.114 (h-/h- pat1.114/pat1.114 leu1.32/leu1.32 ade6M210/ade6M216) asynchronous cells were grown in Minimal Medium (MM) supplemented with 0,1 g/l leucine at 25°C up to a A595 = 0.8. Synchronous meiosis of diploid pat1.114 cells was induced as described [6].

### Preparation of mononucleosomal DNA

Mononucleosomal DNA for microarray hybridization and sequencing was isolated as described [6]. The amount of Zymolyase 20 T used to prepare spheroplasts was optimized experimentally for each *S. pombe* strain and for the different physiological conditions to generate a 80:20 ratio of mononucleosomes to dinucleosomes, as described [4]. The following amounts of Zymolyase were added to the cell suspension in 10 ml of sorbitol–Tris buffer: Meiosis 0 h (10 mg), meiosis 3 h (40 mg), meiosis 5 h (65 mg), mitosis (40 mg), *atf1*Δ (8 mg), *pcr1*Δ (15 mg).

### Microarray analysis of transcription

For quantitative transcriptional analyses, Affymetrix GeneChip *S. pombe* 1.0FR tiling microarrays were used. Target labelling preserving the original polarity of RNAs was performed following the instructions of the GeneChip whole-transcript sense target-labelling assay manual from Affymetrix. Quantitative measurement of strand-specific differential transcription in Additional file 5: Table S1, Additional file 7: Table S2, Additional file 8: Table S3, Additional file 11: Table S4 and Additional file 12: Table S5 are indicated as the log2 value of the averaged hybridization signal from the probes spanning every ORF in the microarray. A detailed description of the method has been previously described [43]. Normalized raw microarray signals without smoothing or denoising are shown in Figures 2, 3, 4, 5, 6 and Additional file 9: Figure S6 (green vertical lines). Hybridization signals from probes mapping to more than one position in the genome were normalized relative to the number of repeats. For all the genomic analyses, we used the *S. pombe* genome version of 23/08/07 and the annotation of 24/02/11 in (http://www.pombase.org) as a reference.

Affymetrix GeneChip *S. pombe* 1.0FR tiling microarrays were hybridized with mononucleosomal DNA from wild type, *atf1*Δ and *pcr1*Δ *S. pombe* strains (Figure 6) following the instructions of the Affymetrix GeneChip whole-transcript double-stranded target-labelling assay manual.

### Sequencing analysis of mononucleosomal DNA and nucleosome depleted regions

Mononucleosomal DNA isolated as described above from haploid and diploid *S. pombe* strains and from cells at 0, 3 and 5 h into meiosis was sequenced in an Illumina Genome Analyzer IIx. 16588557 to 35703552 single reads 36 or 40 nucleotides long, depending on the sample, representing an average genome coverage ranging from 46- to 177-fold were aligned to the *S. pombe* reference genome described above. The alignment of reads generated two peaks (one on each strand) corresponding to the boundaries of each nucleosome. We used the smoothed signal generated by using the multilevel 1-D biorthogonal wavelet decomposition/reconstruction tool implemented in the Matlab “Wavelet Toolbox” to calculate the average spacing between boundary peaks for individual nucleosomes.

This parameter (which was estimated for every independent experiment) defined the distance that the individual profiles from each DNA strand had to be shifted to converge and define the midpoint position of each nucleosome. The resulting combined profile was wavelet-smoothed to generate the final nucleosome positioning profile. Comparison of the raw and wavelet-processed data in Additional file 1: Figure S1 shows that the mathematical modification of the raw sequence data was minimal. The wavelet-smoothed signal facilitated the straightforward detection of specific nucleosomes (−1, +1, central and terminal) relative to the transcription initiation or termination sites. Alignment to the +1 nucleosome (Figure 1) has been used in previous studies [68, 69] and generates sharper profiles than alignment to the transcription start site (TSS) (Additional file 3: Figure S3A).

The sequencing coverage for every nucleotide was divided by the average genomic coverage to normalize the different experiments. The Pearson Correlation Coefficient between all possible combinations of the complete raw sequencing datasets of mitosis and meiosis at 0, 3 and 5 hours, ranged between 0.74 and 0.88. We defined nucleosome-depleted regions (NDRs) as regions spanning at least 150 nucleotides (corresponding to the eviction of at least one nucleosome) with a normalized sequence coverage lower than 0.4. The resulting population of NDRs was largely coincident with those detected previously using tiling microarrays [6].

### Sequence motif analysis

To identify DNA motifs, sequences from specific groups of NDRs were extracted and used as input for the MEME (Multiple EM for Motif Elicitation) algorithm [35] using the same settings as described [36], except that we used as a background set a fifth-order Markov model representing possible nucleotide pentuplets in all *S. pombe* 5′ NDRs.

### Data access

The genomic data described in this work can be accessed from a searchable genome browser at http://genomics.usal.es/cgi-bin/gb2/gbrowse/Sp_nucdyn. All sequencing and microarray data are deposited in the Gene Expression Omnibus (GEO) database under the accession number GSE41773.

## Electronic supplementary material

Additional file 1: Figure S1: Comparison of nucleosomal profiles from raw and processed mononucleosomal sequencing data. The top profile represents an example of the individual nucleotide coverage after aligning sequence reads directly onto the *S. pombe* reference genome. The bottom profile represents the same data after wavelet smoothing of the raw signal, as described in Methods. (PDF 60 KB)

Additional file 2: Figure S2: Comparison of nucleosomal profiles generated by MNase_Seq and by Southern hybridization. (A) Nucleosomal profile of the *rad16* gene as detected by sequencing of mononucleosomal DNA. The 3.4 kb Spe I restriction fragment analyzed in B, the hybridization probe (green), the position of some size markers and the exons and introns of the *rad16* gene (blue) are indicated, (B) Southern hybridization analysis of the same region after chromatin digestion with increasing amounts of Micrococcal Nuclease (MNase) followed by Spe I digestion, electrophoresis, blotting and hybridization to an end-terminal probe (green bar) was done as described in Reference [6]. Introns in the *rad16* gene are not indicated. The resulting profile of positioned nucleosomes (yellow circles in A and ovals in B) was identical in both cases. (PDF 934 KB)

Additional file 3: Figure S3: Comparative nucleosomal profiles of *S. pombe* and *S. cerevisiae.* The aggregated nucleosomal profiles of approximately 4000 *S. pombe* and *S. cerevisiae* genes were aligned to the midpoint position of the +1 nucleosome (+1 N) and to the transcription start site (TSS) (A), to the −1 nucleosomes (−1 N) (B), and to the terminal nucleosomes (TN) or to the transcription termination sites (TTS) (C). Nucleosome profiles for *S. cerevisisae* were represented from the sequencing data of Tsui et al. [70]. The coordinates of TSS and TTS have been reported by Lee et al. [3]. The marked differences between the TTS and TN profiles in the two yeasts is due to the variable distance between the TTS and the midpoint of the terminal nucleosome (TN) that severely diminishes the sharpness of the nucleosomal profile when the TTS is used as a reference for the alignment. (PDF 163 KB)

Additional file 4: Figure S4: Nucleosome positioning over long genes. Nucleosome positioning (meiosis at 3 h) is maintained along the *ppk19* (5345 bp), *utp10* (5281 bp) and *alm1* (5392 bp) genes. Solid and open bars represent translated and non-translated fractions of the transcripts, respectively. (PDF 56 KB)

Additional file 5: Table S1: Gene expression (log2) during mitosis and meiosis at 0 h, 3 h and 5 h. (XLS 572 KB)

Additional file 6: Figure S5: Nucleosome profile of transcribed and intergenic regions in *S. pombe* in mitotic and meiotic cells. The nucleosome profile of the same *S. pombe* genes as described in Figure 1 were aligned relative to the midpoint position of the +1 (+1 N) (A), -1 (−1 N) (B), central (CN) (C) and terminal (TN) (D) nucleosomes of each transcription unit. Diagrams represent the relative nucleosome occupancy profiles from exponential mitotic diploid *pat1.114* cells (blue) and from a synchronous culture of diploid *pat1.114* cells at 3 hours into meiosis (red). The small difference in the alignment relative to TN is probably due to the presence of meiosis-specific NDRs, which are absent in mitotic cells (See text for details). (PDF 236 KB)

Additional file 7: Table S2: Size and genomic coordinates of constitutive, meiosis- and mitosis-specific NDRs. (XLS 269 KB)

Additional file 8: Table S3: List of 352 genes overexpressed more than 4-fold in meiosis at 0 h relative to mitosis; in meiosis at 3 h relative to 0 h; and in meiosis at 5 h relative to 3 h. (XLS 60 KB)

Additional file 9: Figure S6: Nucleosome organization and differential gene expression. The nucleosome profile of the *meu14* (A), *mcp3* (B) and *SPCC11E10.09c* (C) genes remains unchanged although they are overexpressed 22.1-fold, 10.7-fold and 14.2-fold in meiosis at 3 h relative to 0 h, respectively. In meiosis at 0 h the three genes are expressed 1.6-fold, 2.3-fold and 2.7-fold above the background. (PDF 250 KB)

Additional file 10: Figure S7: Distribution of transcription factors binding motifs at NDRs associated with meiosis-specific and stress-response genes. (A) Distribution of sequence motifs identified by MEME in the NDRs of 82, 88 and 41 genes specifically expressed during meiosis at 0 h, 3 h and 5 h. The distribution of motifs corresponding to the binding sites for the transcription factors Ste11 and Mei4 (red line) is shown relative to the aggregated nucleosome profiles (black line). Binding sites for Ste11 are overrepresented in the NDRs of genes specifically expressed in meiosis at 0 h while those for Mei4 are overrepresented in genes expressed at 3 h and 5 h. (B) CRE and CRE variant sites are overrepresented in the NDRs of genes overexpressed under oxidative stress. The distribution of motifs was calculated as described [16]. (PDF 363 KB)

Additional file 11: Table S4: Differential expression of the 22 genes whose 5′ NDR depends on Atp1/Pcr1. (XLS 26 KB)

Additional file 12: Table S5: Differential expression of the genes downstream from the 148 Atf1/Pcr1 major binding sites *in vivo*. (XLS 48 KB)
