# Supplementary figures and images for: Clustered regulatory elements at nucleosome-depleted regions punctuate a constant nucleosomal landscape in Schizosaccharomyces pombe
Source: BMC Genomics. 2013 Nov 21;14(1):813. doi: 10.1186/1471-2164-14-813 (PMC4046669; doi:10.1186/1471-2164-14-813)

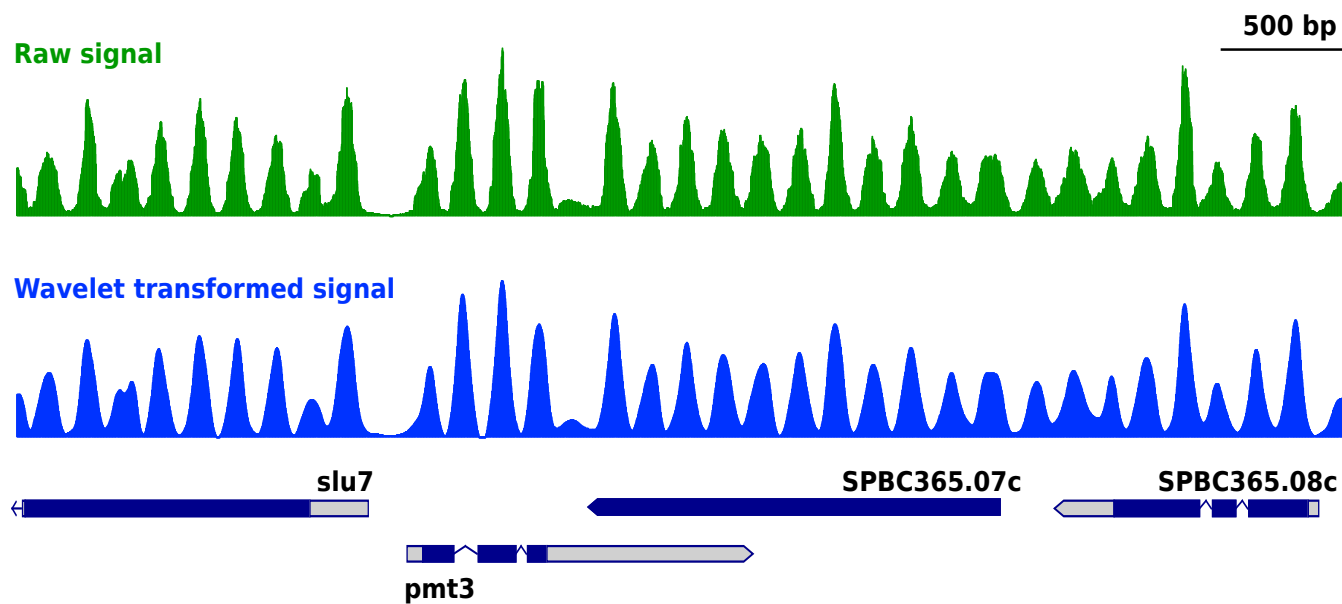

**Supplementary Figure 1**

Supplement: Supplementary file 1 — Additional file 1: Figure S1: Comparison of nucleosomal profiles from raw and processed mononucleosomal sequencing data. The top profile represents an example of the individual nucleotide coverage after aligning sequence reads directly onto the S. pombe reference genome. The bottom profile represents the same data after wavelet smoothing of the raw signal, as described in Methods. (PDF 60 KB) [file 12864_2013_5520_MOESM1_ESM.pdf]

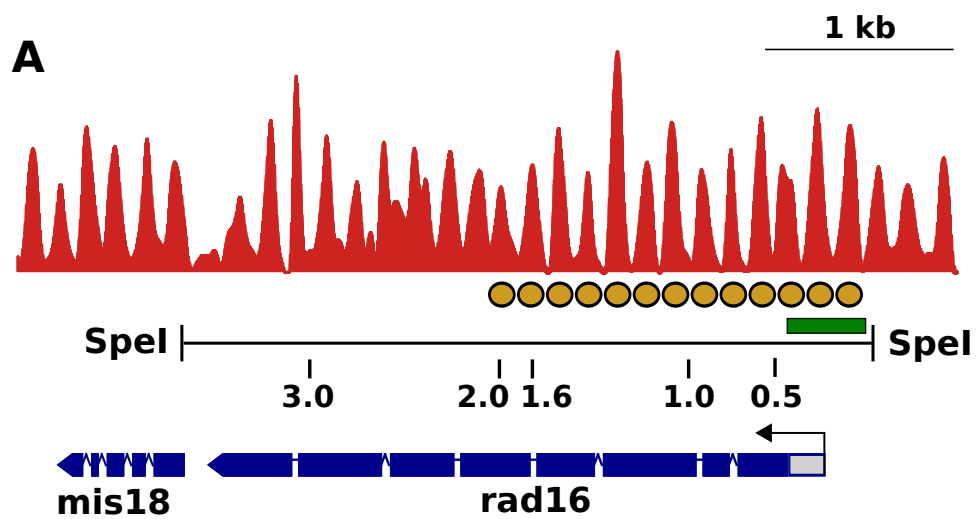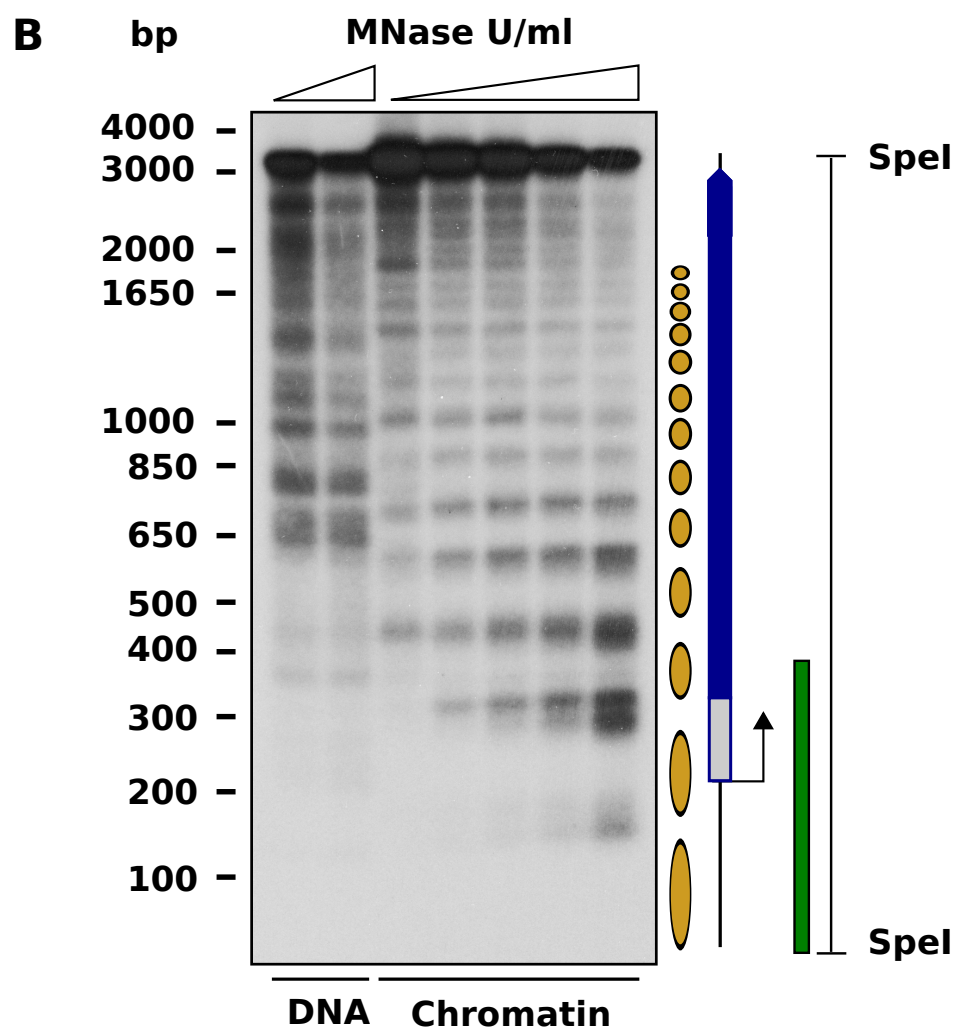

Supplementary Figure 2

Supplement: Supplementary file 2 — Additional file 2: Figure S2: Comparison of nucleosomal profiles generated by MNase_Seq and by Southern hybridization. (A) Nucleosomal profile of the rad16 gene as detected by sequencing of mononucleosomal DNA. The 3.4 kb Spe I restriction fragment analyzed in B, the hybridization probe (green), the position of some size markers and the exons and introns of the rad16 gene (blue) are indicated, (B) Southern hybridization analysis of the same region after chromatin digestion with increasing amounts of Micrococcal Nuclease (MNase) followed by Spe I digestion, electrophoresis, blotting and hybridization to an end-terminal probe (green bar) was done as described in Reference [6]. Introns in the rad16 gene are not indicated. The resulting profile of positioned nucleosomes (yellow circles in A and ovals in B) was identical in both cases. (PDF 934 KB) [file 12864_2013_5520_MOESM2_ESM.pdf]

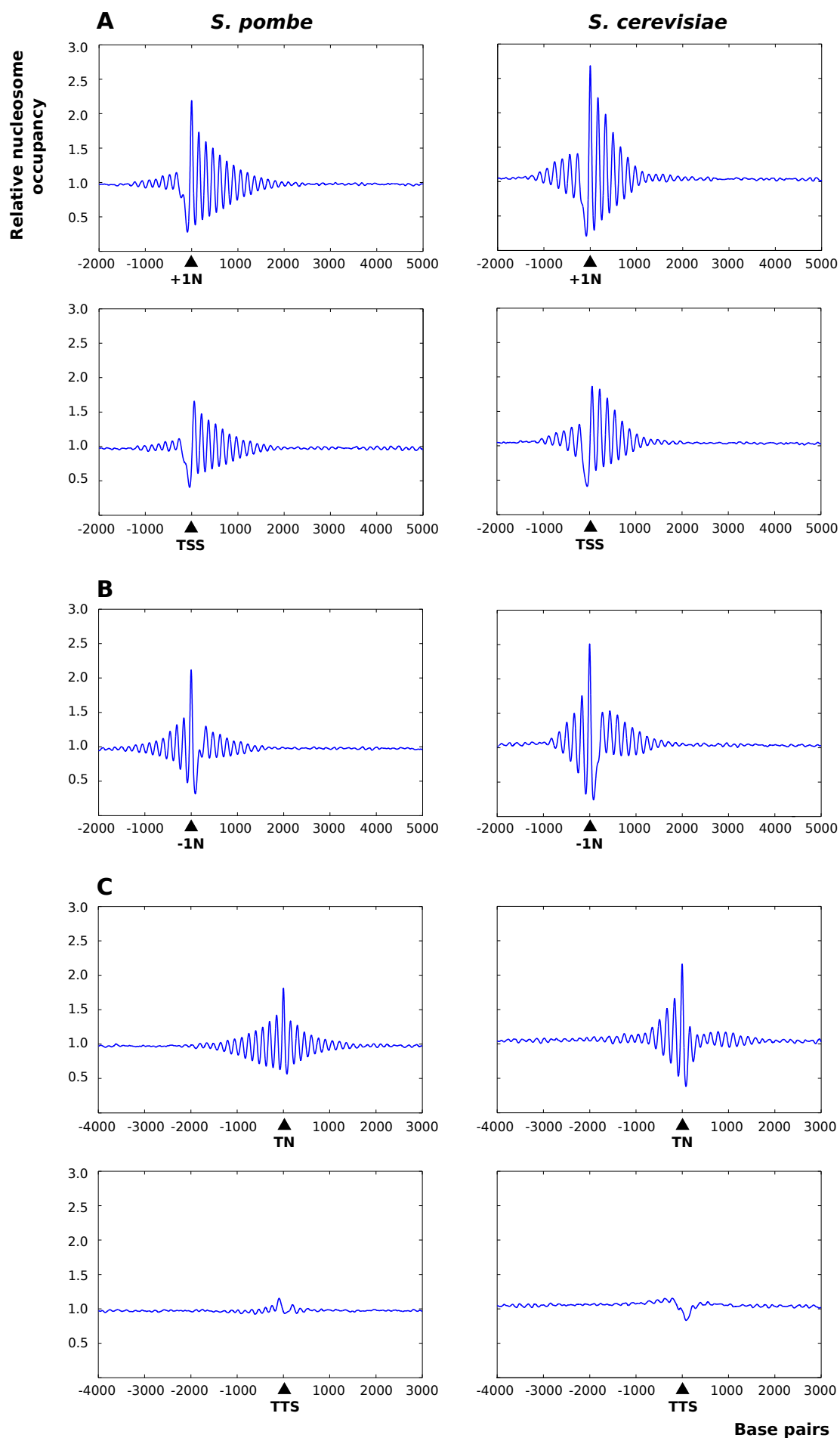

**Supplementary Figure 3**

Supplement: Supplementary file 3 — Additional file 3: Figure S3: Comparative nucleosomal profiles of S. pombe and S. cerevisiae. The aggregated nucleosomal profiles of approximately 4000 S. pombe and S. cerevisiae genes were aligned to the midpoint position of the +1 nucleosome (+1 N) and to the transcription start site (TSS) (A), to the −1 nucleosomes (−1 N) (B), and to the terminal nucleosomes (TN) or to the transcription termination sites (TTS) (C). Nucleosome profiles for S. cerevisisae were represented from the sequencing data of Tsui et al. [70]. The coordinates of TSS and TTS have been reported by Lee et al. [3]. The marked differences between the TTS and TN profiles in the two yeasts is due to the variable distance between the TTS and the midpoint of the terminal nucleosome (TN) that severely diminishes the sharpness of the nucleosomal profile when the TTS is used as a reference for the alignment. (PDF 163 KB) [file 12864_2013_5520_MOESM3_ESM.pdf]

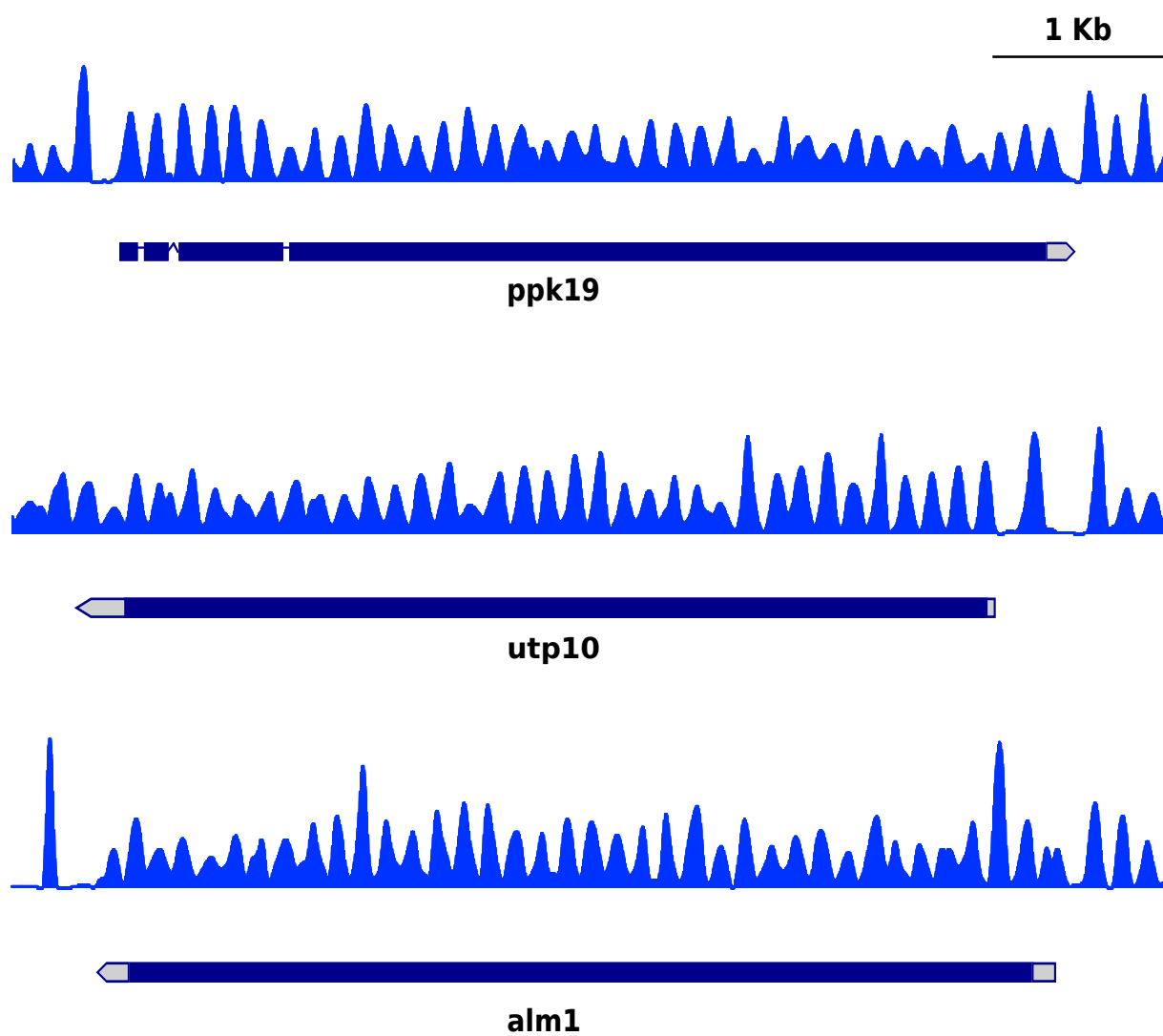

Supplementary Figure 4

Supplement: Supplementary file 4 — Additional file 4: Figure S4: Nucleosome positioning over long genes. Nucleosome positioning (meiosis at 3 h) is maintained along the ppk19 (5345 bp), utp10 (5281 bp) and alm1 (5392 bp) genes. Solid and open bars represent translated and non-translated fractions of the transcripts, respectively. (PDF 56 KB) [file 12864_2013_5520_MOESM4_ESM.pdf]

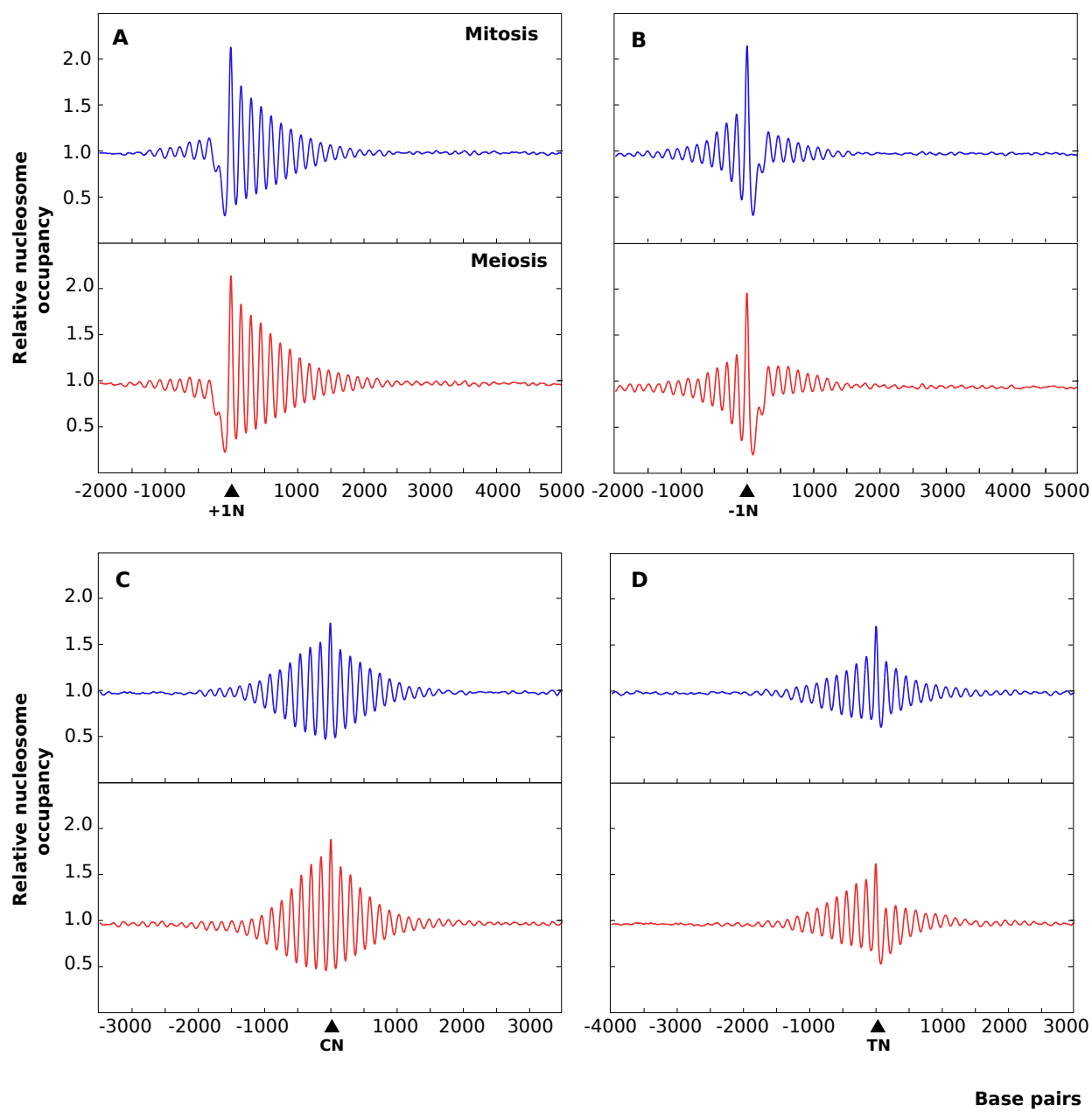

**Supplementary Figure 5**

Supplement: Supplementary file 6 — Additional file 6: Figure S5: Nucleosome profile of transcribed and intergenic regions in S. pombe in mitotic and meiotic cells. The nucleosome profile of the same S. pombe genes as described in Figure 1 were aligned relative to the midpoint position of the +1 (+1 N) (A), -1 (−1 N) (B), central (CN) (C) and terminal (TN) (D) nucleosomes of each transcription unit. Diagrams represent the relative nucleosome occupancy profiles from exponential mitotic diploid pat1.114 cells (blue) and from a synchronous culture of diploid pat1.114 cells at 3 hours into meiosis (red). The small difference in the alignment relative to TN is probably due to the presence of meiosis-specific NDRs, which are absent in mitotic cells (See text for details). (PDF 236 KB) [file 12864_2013_5520_MOESM6_ESM.pdf]

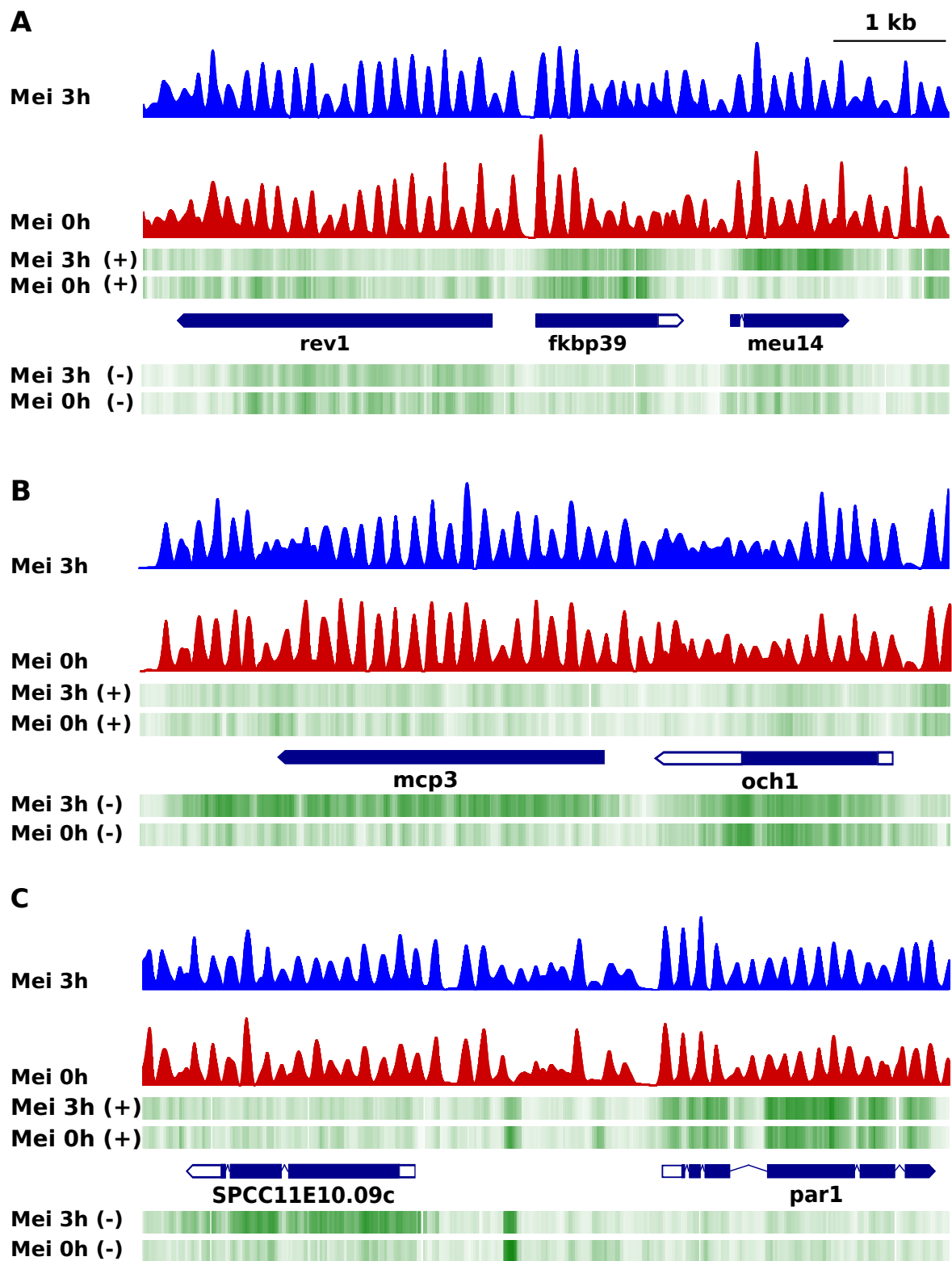

Supplementary Figure 6

Supplement: Supplementary file 9 — Additional file 9: Figure S6: Nucleosome organization and differential gene expression. The nucleosome profile of the meu14 (A), mcp3 (B) and SPCC11E10.09c (C) genes remains unchanged although they are overexpressed 22.1-fold, 10.7-fold and 14.2-fold in meiosis at 3 h relative to 0 h, respectively. In meiosis at 0 h the three genes are expressed 1.6-fold, 2.3-fold and 2.7-fold above the background. (PDF 250 KB) [file 12864_2013_5520_MOESM9_ESM.pdf]

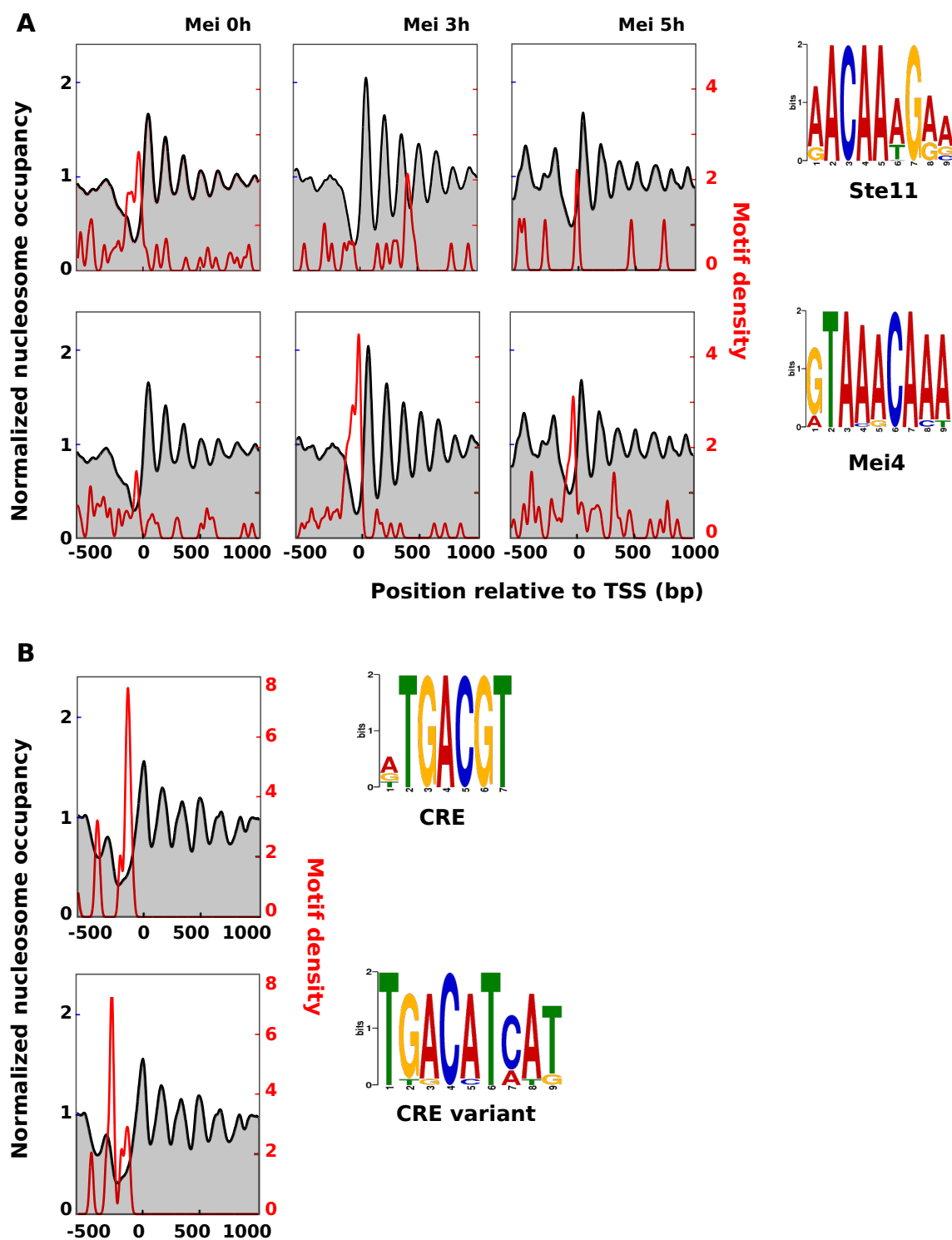

Supplementary Figure 7

Supplement: Supplementary file 10 — Additional file 10: Figure S7: Distribution of transcription factors binding motifs at NDRs associated with meiosis-specific and stress-response genes. (A) Distribution of sequence motifs identified by MEME in the NDRs of 82, 88 and 41 genes specifically expressed during meiosis at 0 h, 3 h and 5 h. The distribution of motifs corresponding to the binding sites for the transcription factors Ste11 and Mei4 (red line) is shown relative to the aggregated nucleosome profiles (black line). Binding sites for Ste11 are overrepresented in the NDRs of genes specifically expressed in meiosis at 0 h while those for Mei4 are overrepresented in genes expressed at 3 h and 5 h. (B) CRE and CRE variant sites are overrepresented in the NDRs of genes overexpressed under oxidative stress. The distribution of motifs was calculated as described [16]. (PDF 363 KB) [file 12864_2013_5520_MOESM10_ESM.pdf]
